# Silver(I) Bromide Phosphines Induce Mitochondrial-Mediated Apoptosis in Malignant Human Colorectal Cells

**DOI:** 10.3390/biomedicines11102794

**Published:** 2023-10-14

**Authors:** Kim Elli Roberts, Zelinda Engelbrecht, Kariska Potgieter, Reinout Meijboom, Marianne Jacqueline Cronjé

**Affiliations:** 1 School of Molecular and Cell Biology, University of the Witwatersrand, Johannesburg 2050, South Africa; elliroberts@outlook.com (K.E.R.);; 2 Research Centre for Synthesis and Catalysis, Department of Chemical Sciences (APK), University of Johannesburg, Johannesburg 2006, South Africarmeijboom@uj.ac.za (R.M.)

**Keywords:** silver(I) bromide phosphine, HT-29, anti-cancer, apoptosis, mitochondria, flow cytometry

## Abstract

Due to its emerging resistance to current therapies, colon cancer remains one of the most difficult types of cancer to treat. Silver, a non-invasive metal, is well-known for its antimicrobial and anti-cancer properties. Two novel silver(I) phosphine complexes, [silver(I) diphenyl-2-pyridylphosphine]Br (**1**) and [silver(I) is 4-(dimethylamino)phenyldiphenylphosphine]Br (**2**), were synthesized and characterized by elemental analysis, infrared spectroscopy, and nuclear magnetic resonance (^1^H, ^13^C, ^31^P). To assess the complexes’ potentials as antiproliferative agents, experiments were conducted on human colorectal cancer cells (HT-29) in vitro. The evaluation involved the analysis of morphological changes, the performance of an alamarBlue^®^ proliferation assay, and the undertaking of flow cytometric analyses to detect mitochondrial alterations. Complex **1** displayed superior selectivity and significant inhibitory effects on malignant HT-29 cells while exhibiting minimal toxicity towards two non-malignant HEK-293 and MRHF cells. Moreover, after 24 h of treatment, complex **1** (IC_50_, 7.49 µM) demonstrated higher efficacy in inhibiting cell proliferation compared with complex **2** (IC_50_, 21.75 µM) and CDDP (IC_50_, 200.96 µM). Flow cytometric studies indicated that complex **1** induced regulated cell death, likely through mitochondrial-mediated apoptosis. Treatment with complex **1** induced morphological changes indicative of apoptosis, which includes membrane blebbing, PS externalization, increased levels of reactive oxygen species (ROS) and mitochondrial membrane depolarization (ΔΨm). These observations suggest that complex **1** targets the mitochondria and holds promise as a novel metal-based anti-cancer therapeutic for the selective treatment of colorectal cancer.

## 1. Introduction

Colorectal cancer remains the third deadliest cancer in the world, affecting both men and women and with mortality rates of 9% and 8%, respectively [1]. Colectomy is the most common approach to remove the infected site if metastasis is not evident. When cancer metastasizes, colectomy is usually combined with chemotherapy [2]. However, colorectal cancer cells present certain abnormalities within the apoptotic pathway which result in resistance to chemotherapy [3].

Metal-based complexes hold promise as applications for the development of chemotherapeutic agents [4]. Cisplatin, a platinum-based drug, has been a powerful and renowned anti-cancer agent against various types of cancers [5]. It is one of the most common treatments for colon cancer, along with 5-fluorouracil [6]. Its mechanism of action involves the ability to interfere with DNA repair mechanisms by linking to purine bases, causing DNA damage, and inducing apoptosis in cancerous cells [5]. However, the emergence of chemoresistance in patients undergoing treatment with cisplatin is a significant issue and it is associated with a variety of harmful side effects, including neurotoxicity and renal insufficiency, which are of great concern [7,8,9].

Silver (Ag) has been used for its antibacterial and antifungal effects for many decades. Silver nanoparticles (AgNPs) have gained recognition as effective antibacterial agents by releasing silver ions that disrupt the bacterial cell membrane. This causes the generation of ROS and hinders ATP synthesis [10,11]. When bound to different ligands, silver exhibits a wide range of characteristics. These include structural diversity, the ability to bind different ratios of ligands to the transition metal or replace them and possessing effective redox and catalytic properties. Among these are silver complexes synthesized with N-heterocyclic carbenes, carboxylates, Schiff bases and phosphines [12,13]. Silver(I) phosphine complexes, specifically, have shown impeding potential as cancer therapeutic agents in vitro. A series of Ag(I) complexes with mixed ligand coordination of diphenyl(p-tolyl) phosphine and thiosemicarbazone derivates showed anti-cancer activity in two breast and one colon cancer cell line with IC_50_ concentrations ranging from 2.2 to 4.6 µM [14]. Ag(I) thiocyanate complexes coordinated with different phosphine ligands have shown a higher degree of activity in breast [15] and esophageal [16,17,18,19,20] cancer cell models than cisplatin. 

Research is slowly validating the capabilities of Ag(I) phosphines and determining their mechanism of action toward cancerous cells. An Ag(I) cyanide–phosphine complex was confirmed to induce apoptosis by the presence of phosphatidylserine (PS) externalization, DNA fragmentation, and nuclear condensation in a malignant esophageal cell line [19]. The same apoptotic features have been observed in breast cancer cells treated with a series of Ag(I) thiocyanate complexes with the addition of cell cycle arrest in the G2/M phase and caspase-9, -8 and -7 cleavage, indicating both extrinsic and intrinsic (also known as mitochondrial-mediated) pathway involvement [15,21]. An Ag(I) complex containing a 3-methoxy-4-hydroxybenzaldehyde thiosemicarbazone and triphenylphosphine ligand system also induced apoptosis in a triple-negative breast cancer cell line, accompanied by a decrease of the mitochondrial membrane potential (ΔΨm) and reactive oxygen species (ROS) production [22]. An Ag(I) thiocyanate 4-methoxyphenyl phosphine also induced apoptosis, which resulted in the disruption of the mitochondrial membrane, the decrease of the ΔΨm, ROS production and caspase-9 cleavage, further implicating the involvement of the mitochondrial-mediated apoptotic pathway [20]. 

Mitochondrial integrity is essential for cell viability and proliferation, especially in the production of ATP and in maintaining homeostatic processes [23,24]. ATP production is linked to the transition of metabolites and the generation of redox molecules, which produce ROS. In typical cellular conditions, various antioxidant pathways function to counteract the harmful effects of ROS and maintain the balance of their redox state. An elevated ROS level has the potential to damage mitochondria, leading to cellular dysfunction and possibly resulting in cell death [25,26]. Cancer cells exhibit several degrees of mitochondrial dysfunction compared with non-cancerous cells. Mitochondrial dysfunction enmeshes the change in energy metabolism from oxidative phosphorylation to glycolysis, leading to reduced levels of ATP and a transition in membrane permeability [26]. The most effective method of influencing cellular redox homeostasis is by increasing mitochondrial ROS levels [25,27]. It has been reported that the biological effects of silver complexes are mitochondrial dysfunction, DNA interaction, induction of elevated ROS levels, and cell wall disruption. Silver complexes can interfere with the redox reactions of the thiol group, which can lead to a blockage of electron transfer, cellular respiration, inactivation of essential enzymes, and binding to DNA via formation of a disulphide bond [9,20,22,25]. The ability to define specific differences between normal and cancerous cells allows the targeting of mitochondria as a potential strategy by which to destroy cancer cells and provide advancement in therapeutic selectivity.

Multiple studies have shown that silver(I) phosphine complexes within the same family suppress cancer cell proliferation and induce apoptosis in various malignant cell lines [15,16,17,18,19,20]. The coordination and geometries of silver(I) phosphine complexes exhibit an extensive number of structural types that can be readily manipulated by varying the ligands and silver salts and their ratios, and thereby encouraging the study of silver(I) chemistry [18].

**Figure 1:**
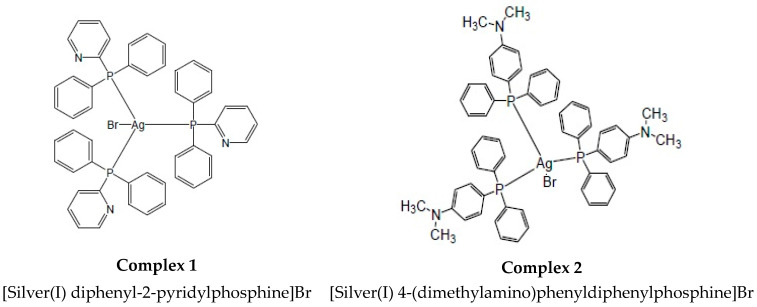
The chemical structures of silver(I) phosphine complexes **1** and **2**.

The purpose of this study was to screen two novel silver(I) mono–dentate phosphine complexes (and their ligands) to determine whether they were selective in effectively decreasing the cellular viability in colorectal cancer cells. In this study, we present the anti-cancer ability of these complexes and their involvement in apoptosis induction and mitochondrial targeting. The complexes were coordinated with an AgBr metal salt with either diphenyl-2-pyridylphosphine or with 4- (dimethylamino)phenyldiphenylphosphine, which are herein referred to as complex **1** and **2**, respectively. Both of these complexes orientate as a tetrahedral geometry with three phosphine ligands surrounding the AgBr salt core (1:3 ratio) (Figure 1).

## 2. Materials and Methods

### 2.1. General

The two complexes studied herein were synthesized at the University of Johannesburg’s Department of Chemical Sciences using chemicals obtained from Sigma-Aldrich (St. Louis, MI, USA). Before being used in biological studies, complexes 1 and 2 were further characterized. Melting points were measured using a melting point apparatus with a microscope. Dr. Edith Antunes performed the elemental analysis at Rhodes University’s Department of Chemistry (Makhanda, South Africa) utilizing a Thermo Flash 2000 series CHNS/O, Organic Elemental Analyzer (Waltham, MA, USA). FT-IR spectra were captured using a PIKE miracle Gate ATR attachment (Madison, WI, USA) on a Bruker Tensor 27 FT-IR spectrometer (Billerica, MA, USA). A Bruker Ultrashield Avance III 400 MHz spectro-meter was used to measure and record nuclear magnetic resonance (NMR) spectra. Spectra for ^1^H-NMR, ^13^C{H}-NMR and ^31^P{H}-NMR were obtained in DMSO-d6 at 400 MHz for ^1^H nuclei, 75 MHz for ^13^C{H} nuclei and 161 MHz for ^31^P{H} nuclei. All chemical shifts were reported in parts per million (ppm), with residual H or C serving as internal references. All ^31^P{H} NMR chemical shifts were compared with an external standard, H_3_PO_4_.

### 2.2. Synthesis of 1:3 [Silver(I) Diphenyl-2-pyridylphosphine]Br (Complex ***1***)

AgBr salt (0.1333 g; 0.71 mmol) was added to diphenyl-2-pyridylphosphine (0.561 g and 2.13 mmol) (**L1**) in acetonitrile (50 mL). The solution was heated overnight under reflux. The hot solution was filtered, and the solvent was reduced to ±10 mL and left to crystalize for 24 h, from which small white needles were isolated. On average, 2 mg of compound was prepared with 80% purity. To exclude possible variations during treatments, all experiments were conducted with the same batch. Melting point: 183–186 °C. Elemental Analysis: Calculated for C_51_H_42_AgBrN_3_P_3_: C, 62.66%; H, 4.33%; N, 4.30%. Found: C, 62.84%; H, 4.37%; N, 4.42%. IR (v/cm^−1^): 1569.99, 1479.34, 1448.83, 1433.24, 1417.92 (v (aromatic, C-H bend, meta), s), 1277.34, 1151.29, (v, C-N), 1092.28, 1046.53, 1028.20, 997.43, 987.26 (v (aromatic, C-H bend, meta), s), 742.47, 720.25, 691.33, 617.77, 521.03 (v (aromatic, C-H bend, ortho), m). ^1^H-NMR (400 MHz, CDCl_3_) δ ppm: 7.140–7.183 (m, aromatic H), 7.251–7.295 (m, aromatic H), 7.337–7.377 (m, aromatic H), 7.476–7.548 (m, aromatic H). ^13^C{H}-NMR (100 MHz, CDCl_3_) δ ppm: 123.100 (s, aromatic C), 128.589 (d, aromatic C), 129.507 (s, aromatic C), 129.731 (d, aromatic C), 133.245 (d, aromatic C), 134.331 (d, aromatic C), 135.872 (d, aromatic C), 150.377 (d, pyridyl C), 159.800 (s, pyridyl C), 160.106 (pyridyl C). ^31^P{H} NMR (161 MHz, CDCl_3_) δ ppm: 3.63 (s). The Supplementary Information File S1 has the inclusion of both IR and NMR spectra.

### 2.3. Synthesis of 1:3 [Silver(I) 4-(Dimethylamino)phenyldiphenylphosphine]Br (Complex ***2***)

AgBr salt (0.1333 g; 0.71 mmol) was added to a solution of 4-(dimethylamino)phenyldiphenylphosphine (0.650 g and 2.13 mmol) (**L2**) in acetonitrile (50 mL). The solution was heated under reflux overnight. The hot mixture was filtered and allowed to evaporate until it reached approximately 10 mL. This solution was left to crystallize for 24 h at room temperature, from which small white needle crystals were isolated. Melting point: 209–211 °C. Elemental Analysis: Calculated for C_60_H_60_AgBrN_3_P_3_: C, 65.29%; H, 5.48%; N, 3.81%. Found: C, 65.94%; H, 5.63%; N, 3.35%. IR (v/cm^−1^): 1596.59, 1543.18, 1511.06 (v (aromatic, C=C aromatic, m), 1477.84, 1445.91, 1432.75 (v (C=C aromatic), m), 1360.98, 1291.93, 1229.74, 1200.46 (v, C-N, s), 1093.15, 1026.00, 998.57, 943.35 (v (aromatic, C-H bend, meta), s), 804.66, 773.41, 742.27, 694.50, 619.56, 607.95 (v (aromatic, C-H bend, ortho), m). ^1^H-NMR (400 MHz, CDCl_3_) δ ppm: 2.971 (s, methyl H), 6.625 (d, 4-(dimethylamino) H), 7.231–7.369 (m, aromatic H). ^13^C{H}-NMR (100 MHz, CDCl_3_) δ ppm: 40.14 (s, methyl C), 112.21 (s, 4-(dimethylamino)phenyl C), 128.46 (d, aromatic C), 129.08 (s, aromatic C), 133.52 (d, aromatic C), 135.23 (d, aromatic C), 135.79 (d, 4-(dimethylamino)phenyl C). ^31^P{H} NMR (161 MHz, CDCl_3_) δ ppm: 1.53 (s). The Supplementary Information File S2 has the inclusion of both IR and NMR spectra.

### 2.4. Preparation of Complexes

Stock solutions (2 mM) of complex **1**, **2** and the ligands (**L1** and **L2**) were dissolved in cell-graded dimethyl sulfoxide (DMSO) (Merck, Darmstadt, Germany). Solutions were heated at 70 °C for 1.5 h until crystals were solubilized. A stock solution of 3.33 mM of CDDP was prepared in 0.9% (*w*/*v*) filtered saline (NaCl) solution followed by heating at 20 °C for 24 h. Stock solutions were kept in the dark at 4 °C and complexes were heated at 70 °C for 30 min prior to experimental treatments. Previous studies using single NMR and IR with related silver(I) complexes have revealed that heating did not change the conformation of the complexes [28]. Control studies using the uncoordinated salt, AgBr, were excluded due to the salt’s poor solubility in DMSO.

### 2.5. Cell Cultures

A non-malignant transformed embryonic kidney (HEK-293) cell line (gifted by the University of KwaZulu-Natal), a non-malignant foreskin fibroblast (MRHF) cell line (Cellonex™ (Johannesburg, South Africa), RSA) and a malignant colorectal adenocarcinoma (HT-29) cell line (Cellonex™, RSA), all of human origin, were used for this study. All cell lines were cultured from stock either in Dulbecco’s modified Eagles medium (DMEM) (Sigma-Aldrich, USA) (HEK-293 and MRHF cells) or in McCoy’s 5A medium (PAN™ Biotech, Aidenbach, Germany) (HT-29 cells). The culture medium was supplemented with 5% heat-inactivated (56 °C, 30 min) fetal calf serum (FCS) (Biowest, Nuaille, France) and 0.6% penicillin/streptomycin/amphotericin B (Lonza, Walkerville, MT, USA). Cells were incubated in a sterile humidified atmosphere at 37 °C, saturated with 5% CO_2_. When cultures attained confluency, cells were washed with Hanks’ balanced salt solution (HBSS) (Gibco^®^ Live Technologies, Swindon, UK) and detached via enzymatic lifting using 1% trypsin/versene solution (Lonza, Walkerville, MT, USA). Cells were collected and seeded for experimentation at either a concentration of 2 × 10^5^ cells/mL in 96-well plates (2 × 10^4^ cells per 100 µL) or in 35 mm tissue culture-treated dishes (6 × 10^5^ cells), in triplicate. Cells were left to adhere for 24 h before treatment.

### 2.6. Cell Treatments and Anti-Proliferative Studies

The malignant HT-29, non-malignant HEK-293 and the MRHF cells were either left untreated (UC, negative control), treated with 1% DMSO (VC, vehicle (mock) control), treated with cisplatin (CDDP, a positive apoptotic control) (Molekula, Dorset, UK), or treated with either complex **1** or **2**. For viability studies and changes in morphology, cells were treated with 100 µM CDDP and 10 µM complex **1** or **2** (including 10 µM of **L1** and **L2** for viability studies only). Cells were left for 24 h and 48 h before fluorescent analyses using the alamarBlue^®^ proliferation assay (Bio-Rad, Watford, UK). Light micrographs were captured to determine changes in morphology after 24 h, using a Zeiss Axiovert 25 inverted light microscope (Carl Zeiss, Oberkochen, Germany) with Zeiss AxioCam MRC camera and Axiovision 3.1 software at 200× magnification. For dose-responsive and apoptogenic studies, only malignant HT-29 cells were further investigated.

To determine inhibitory maximal concentrations of 50% (IC_50_) after 24 h and 48 h, the cells were treated with complexes 1 or 2 at a final concentration of 20 µM per well with subsequent serial dilutions of 10 µM, 5 µM, 2.5 µM, 1.25 µM and 0.625 µM. In addition, cells were also treated with a final concentration of 200 µM CDDP per well with subsequent serial dilutions of 100 µM, 50 µM, 25 µM, 12.5 µM and 6.25 µM. Cells were then left for 24 h and 48 h before analyses using the alamarBlue^®^ proliferation assay. After incubation, 10 µL of alamarBlue^®^ dye was added to the 100 µL of differentially treated cells, which were then incubated in the dark for 2 h at 37 °C. The fluorescence was then measured using a VICTOR^®^ Nivo™ multimode plate reader (excitation at 530/30 nm: emission at 580/20 nm) (PerkinElmer, Waltham, MA, USA). To standardize background fluorescence, a negative control of an amount of 100 µL cell-free culture media was included. The percentage viabilities were expressed with respect to the 1% DMSO vehicle control.

For the apoptogenic analyses, McCoy’s 5A medium was removed from seeded culture dishes and HT-29 cells were treated with 10 µM and 20 µM of complex **1** or **2**. Controls were included and cells were additionally treated with 200 µM of CDDP instead of 100 µM as mentioned previously. Cells were then incubated for 24 h before experimental analyses.

### 2.7. Analysis of Cell Death

To determine the potential mode of cell death the Muse^®^ Annexin V and Dead Cell kit (Luminex Corporation, Austin, TX, USA) was used. The manufacturer’s instructions were followed with limited changes. A total cell density of 3 × 10^5^ cells/mL was used for analyses. Cells were washed with pre-warmed 1× phosphate-buffered saline (PBS; 37 °C) and resuspended in 50 µL 1× assay buffer. Fifty microliters of Annexin V and Dead Cell reagent were then added to each sample, followed by incubation (RT for 20 min in the dark), after which 5000 events were recorded on the Muse^®^ cell analyzer. cells were statistically analyzed using Muse^®^ Software V1.4.0.0 by following the Guava^®^ Muse^®^ Annexin V and Dead Cell kit user’s guide. For compensation and gating, untreated control cells were gated to a threshold that excluded any cellular debris. Data were analyzed and dot plots were constructed.

### 2.8. Change in Mitochondrial Membrane Potential (ΔΨm)

To measure the change in mitochondrial membrane potential and cellular plasma permeabilization, the Muse^®^ MitoPotential kit (Luminex Corporation, Austin, TX, USA) was used. The manufacturer’s instructions were followed with minimal changes. A total cell density of 3 × 10^5^ cells/mL was used for analysis. Before labeling, a working solution was prepared by diluting the Muse^®^ MitoPotential dye (1:1000) with 1× assay buffer. Differentially treated cells were washed with pre-warmed PBS and collected at 300× *g* for 4 min. Samples were resuspended in 50 µL 1× assay buffer. Forty-seven and a half microliters of the working solution were added to this and vortexed. Samples were incubated for 20 min in a 37 °C CO_2_ incubator, after which 1.25 µL of Muse^®^ MitoPotential 7-AAD reagent was added to each sample. Cells were incubated for 5 min in the dark. A total of 5000 events were recorded on the Muse^®^ Cell Analyzer. Cells were statistically analyzed using Muse^®^ Software V1.4.0.0 by following the Guava^®^ Muse^®^ MitoPotential kit user’s guide. Untreated control cells were gated to a threshold that excluded any cellular debris and corresponding data were represented as dot plots.

### 2.9. Quantification of Reactive Oxygen Species (ROS)

To quantify superoxide radicals in HT-29 cells undergoing oxidative stress, the flow cytometric Muse^®^ Oxidative Stress kit (Luminex Corporation, Austin, TX, USA) was used. The manufacturer’s instructions were followed with limited changes. An intermediate reagent was prepared by diluting (1:100) 2 µL of Muse^®^ Oxidative stress stock solution with 198 µL 1× assay buffer (provided in the kit). To obtain a working solution, the intermediate solution was diluted (1:80) with 1× assay buffer. Before labeling, differentially treated cells were washed with 500 µL pre-warmed PBS and collected at 300× *g* for 4 min. Samples were resuspended in 60 µL 1× assay buffer. One hundred and ninety microliters of the working solution were added to the samples and incubated for 30 min at 37 °C in the dark. A total of 3000 events were recorded on the Muse^®^ Cell Analyzer. Cells were statistically analyzed using Muse^®^ Software V1.4.0.0 by following the Guava^®^ Muse^®^ Oxidative Stress kit user’s guide. Untreated control cells were gated a threshold that excluded any cellular debris and corresponding data were represented as histograms.

### 2.10. Statistical Analyses

Microsoft Office Excel with a two-tailed heteroscedastic Student’s *t*-test was used to analyze the data obtained. Errors bars represent the standard mean of error (±SEM) and the *p* value was identified as significant at * 0.05 and highly significant at ** 0.01 and *** 0.001, relative to the vehicle control (1% DMSO). The number of independent biological and technical repeats are represented by *n* in the figure legends.

## 3. Results and Discussion

### 3.1. Viability Studies and Morphological Changes

**Figure 2:**
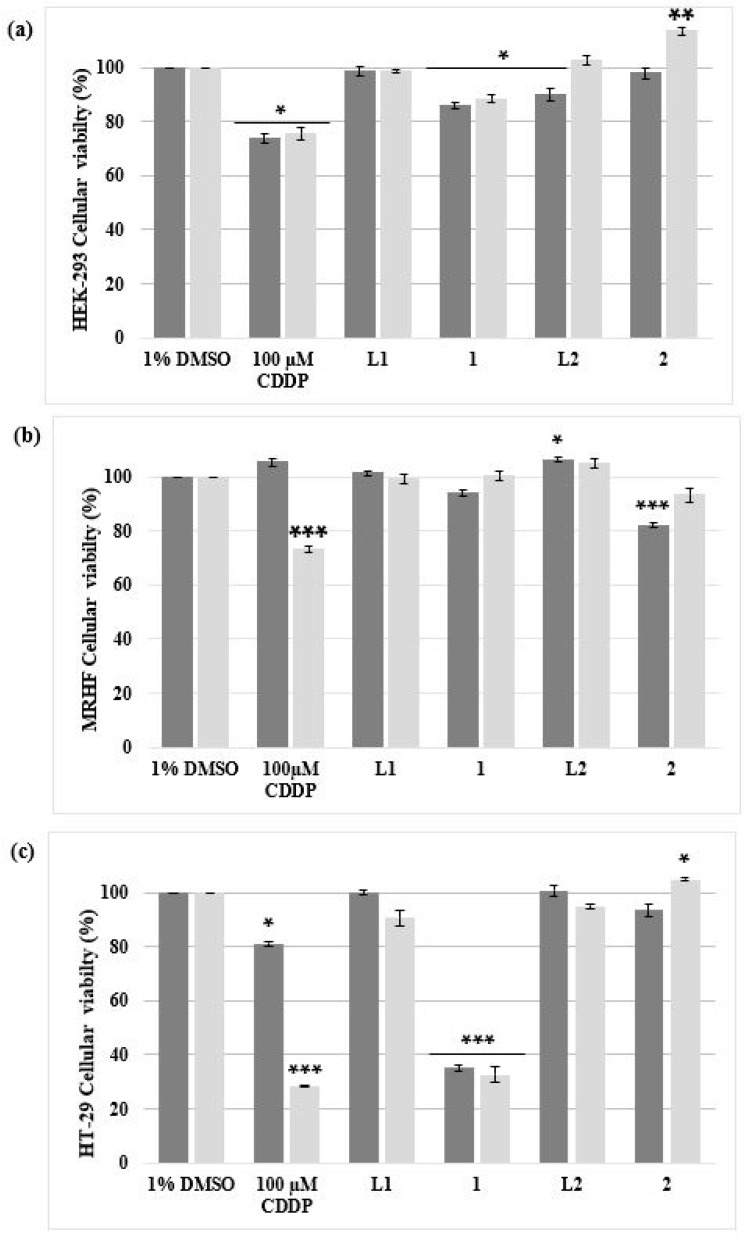
The percentage cell viability of two non-malignant (**a**) HEK-293 and (**b**) MRHF cell lines and (**c**) one malignant HT-29 cell line analyzed using an alamarBlue^®^ proliferation assay. The cells were treated for 24 h (dark grey) and 48 h (light grey) with 10 µM of complex **1** and **2**, including their ligands **L1** and **L2** dissolved in DMSO (vehicle control; VC). Cells treated with 100 µM CDDP, a positive apoptotic control, were also included. The ± SEM (*n* = 3) are represented as error bars. Asterisks indicate significant differences between treatments and 1% DMSO (* *p* < 0.05; ** *p* < 0.01 and *** *p* < 0.001).

To correctly identify a drug with anti-cancer activity, it is important that the complex/es under investigation display selectivity by showing a low degree of toxicity in normal, non-malignant healthy cells and at the same time be toxic to malignant cells. In this study, both non-malignant HEK-293 and MRHF cell models were exposed to the same conditions as the malignant HT-29 cells (Figure 2). Singh et al. (2018) treated HEK-293 cells with increasing concentrations of CDDP and a dose-dependent relationship was observed; as the concentration of CDDP increased, the viability of the HEK-293 cells decreased [29]. In this study, viability remained above 70% at 100 µM treatment of CDDP, after 48 h (Figure 2a). When the HEK-293 cells were treated with complexes **1** or **2**, minimal toxicity was observed after 48 h, with viabilities of 88.40% and 113.49% respectively. Similar viabilities were observed in MRHF cells treated with complexes **1** (100.42%) or **2** (93.27%). Due to the significantly high cellular viability observed, dose-responsive studies on HEK-293 and MRHF cells were not included. Instead, only screening experiments (Figure 2a,b) were conducted to determine whether complexes **1** and **2** were selective for HT-29 cells and were non-toxic to non-malignant healthy cells. Previous studies confirm this low degree of toxicity in healthy cells by Ag(I) phosphine complexes. Engelbrecht et al., (2018a) observed a significant decrease in viability in treated SNO esophageal cancer cells after 24 h, while the non-cancerous cells, HDF-a and HEK-293, were less affected, with viabilities of 88.22% and 74.11%, respectively [20].

HEK-293, MRHF and HT-29 cells treated with the respective ligands (**L1** and **L2**) showed minimal cytotoxicity (Figure 2). Cellular viabilities remained above 85% after 24 h and 48 h treatments. Similar results were observed in findings from a previous study [20]. In addition, a series of AgNO_3_ phosphine complexes, coordinated either in a 1:1, 1:2 or 1:3 manner with a PPh_3_ ligand, derived from the same family of complexes used in the above cytotoxicity studies, was found to be more toxic toward malignant SNO cells than the non-malignant HDF-a cells [30]. Ten micromolar of the ligand, triphenylphosphine, was evaluated against the cells and minimal toxicity was observed, with viabilities higher than 88%. It has been noted that transition metals carrying various ligands result in structures with catalytic abilities. The lipophilic effect of phosphine ligands assists complexes across the cytoplasmic membrane [31]. In general, the interaction of the ligands with the functional Ag(I) complex appears to be the reason for their anti-cancer properties. Ligands derived from mono- and bidentate phosphine ligands have attracted much attention when coordinated with Ag due to these properties [31,32]. Danmak et al. (2020) screened a series of Ag complexes for their antiproliferative activities against human cancer cells. Ag complexes 5 and 10, which are composed of lipophilic phosphine ligands, exhibited high activity. The average IC_50_ values for these complexes against colon (HCT-15) cells were determined to be 2.1 µM and 3.4 µM, respectively [32]. It is believed that the toxicity towards malignant cells is reliant on the type of ligand, as this could contribute to the stability of the complex as well as to the hydrophilic–lipophilic character [31,32,33,34,35,36].

**Figure 3:**
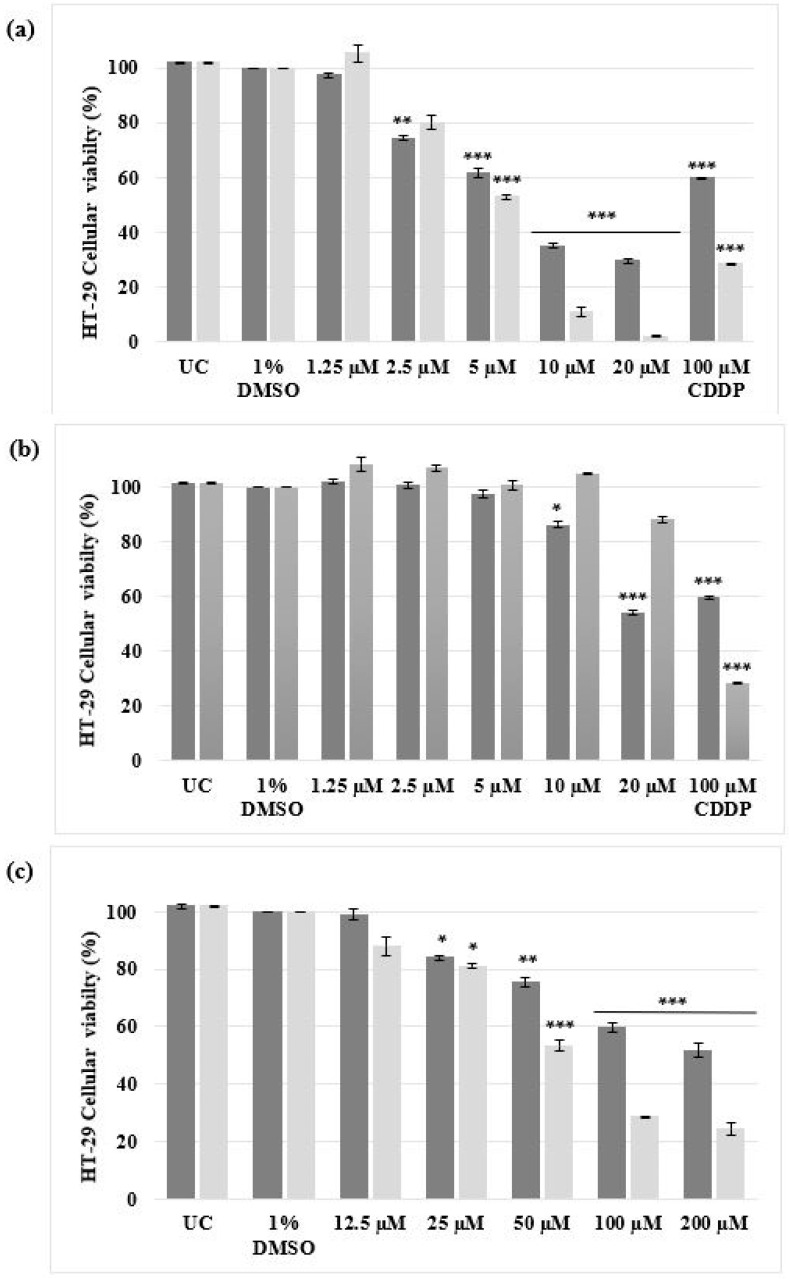
Dose-responsive studies of malignant HT-29 cells determined using the alamarBlue^®^ proliferation assay. The cells were either left untreated or treated with 1% DMSO (VC), a range of concentrations (1.25 µM to 20 µM) of (**a**) complex **1**, (**b**) complex **2** and (**c**) a range of concentrations (12.5 µM to 200 µM) of CDDP for 24 h (dark grey) and 48 h (light grey). The ± SEM (*n* = 3) are represented as error bars. Asterisks indicate significant differences between treatments and 1% DMSO (* *p* < 0.05; ** *p* < 0.01 and *** *p* < 0.001).

When the HT-29 cells were treated, a higher degree of cytotoxicity was identified for complex **1** than complex **2**, shown in Figure 2b. After 48 h, cells treated with complex **1** and **2** resulted in viabilities of 32.58% and 104.92% respectively. Complex **1** cytotoxicity (32.58%) was comparable to CDDP, as the viability after 48 h was 28.50%. The viability of CDDP and complex **1**-treated HT-29 cells decreased significantly (*p* < 0.001) after an additional 24 h of treatment, while cells treated with complex **2** recovered. Following cellular viability studies, dose–response curves (represented as bar graphs) were determined for the HT-29 malignant cells only (Figure 3). After 24 h and 48 h of treatment, both CDDP and complex **2** produced IC_50_ values above 20 µM (Table 1). Complex **1** produced IC_50_ concentrations of 7.49 µM and 5.92 µM after 24 h and 48 h, respectively, as depicted in Figure 3. When considering the structure of the silver(I) complexes, it appears that the phosphine ligands affect the complex’s antiproliferative efficacy, as it is the only element that distinguishes complex 1 from complex **2**. In a study undertaken by Bresciani et al. (2022), IC_50_ values were determined for A549 (human lung carcinoma) and A2780 (human ovarian carcinoma) cells treated with a gold(I) phosphine complex, [AuCl(PPh_3_)] [37], an analog to silver(I) phosphine complexes [36]. IC_50_ values of 18.6 µM (A549 cells) and 12.6 µM (A2780 cells) were obtained, respectively. However, the IC_50_ values obtained for CDDP exceeded 20 µM for both cell lines. Studies have revealed that some transition metal complexes have a higher antiproliferative effect on malignant cells than CDDP [16,17,18,19,20,31,38,39].

**Figure 4:**
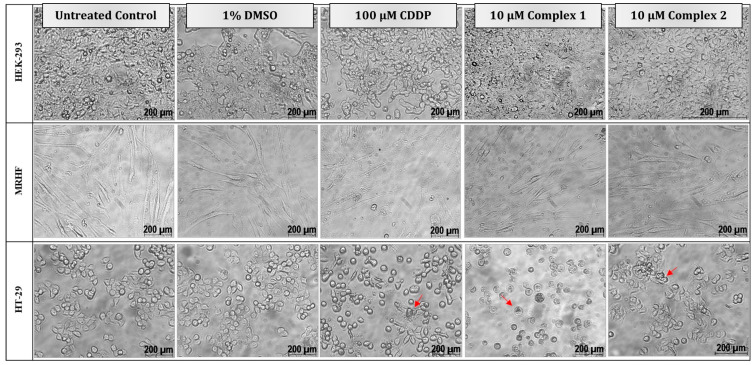
Morphological changes in two non-malignant, HEK-293 (**top**) and MRHF (**middle**), cell lines and one malignant, HT-29 (**bottom**), cell line induced by differential treatments after 24 h. Cells were either left untreated or treated with 1% DMSO (vehicle control), 100 µM CDDP (positive apoptotic control) or treated with 10 µM complex **1** and **2**, respectively. Typical morphological changes to cells diagnostic of apoptosis, such as cell shrinkage, rounding and apoptotic blebbing and bodies, are indicated by the red arrows. Light micrographs were taken with a Zeiss Axiovert 25 inverted light microscope using Axio Vision 3.1 software at 200× magnification.

Morphological studies were conducted on both the non-malignant and malignant cell lines (Figure 4). Cells treated with the vehicle, 1% DMSO, resembled the respective untreated control cells, with no effect on cellular viability (Figure 2). No significant morphological changes were observed for treatments in the HEK-293 and MRHF cells, as they remained confluent and viable. CDDP-treated HT-29 cells appeared to be ‘round’ with the presence of apoptotic bodies (indicated by red arrows). In the HT-29 cells, complex **1** displayed characteristics comparable to apoptotic characteristics such as membrane blebbing and cellular rounding [40]. A less cytotoxic effect was observed when the HT-29 cells were treated with complex **2**, as minimal ‘rounding’ was observed after 24 h.

### 3.2. Confirmation of Apoptotic Cell Death and Mitochondrial Targeting

It should be emphasized that cell viability and morphological studies were conducted using 10 µM treatments of complex **1** and **2** and 100 µM of CDDP. However, to study the complete cell death effect, a concentration of 20 µM for complex **1** and **2** and 200 µM for CDDP was used for the flow cytometry assays, as both complex **1** and CDDP produced similar cell death profiles after 48 h (Figure 1). In addition, the IC_50_ value for CDDP was 200.96 µM. The assays used in this study focus on the mechanisms of cell death, therefore a higher concentration (20 µM) of the silver(I) phosphine treatments allows for a clear representation of the possible cell death mechanisms affected, as cellular viability was less than 50%.

To confirm whether the decrease in cellular viability and morphological changes were caused by the induction of apoptosis, HT-29 cell populations were quantitatively measured using the Muse^®^ Annexin V and Dead Cell assay. This kit can distinguish cells that are still alive, undergoing early or late apoptosis or necrosis. When a cell is undergoing apoptosis, the plasma membrane externalizes, exposing the PS to which Annexin V can bind and consequently be monitored via flow cytometry.

Gil-Moles et al., (2023) revealed that Ag complexes containing PPh_3_ enhanced cytotoxicity in lung (A549), HeLa, and Jurak cells. These complexes exhibited the ability to induce cell death by activating the mitochondrial apoptotic pathway. The cells were cultured for 24 h with 10 µM of the respective compounds and annexin VDY634, which specifically binds to phosphatidylserine on the surface of apoptotic cells. The percentage of apoptotic cells among the Jurkat cells was 82.3% and 60.2%, respectively [37].

**Figure 5:**
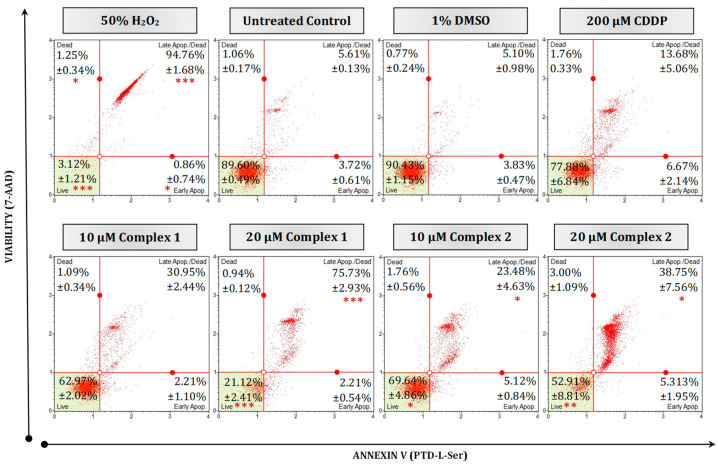
Mode of cell death in malignant HT-29 cells quantified using a Muse^®^ Annexin V and Dead Cell kit. Representative flow cytometry dot plots are presented as 50% H_2_O_2_ (positive necrotic control), untreated, treated with 1% DMSO, 200 µM CDDP, 10 µM and 20 µM of complexes **1** and **2** for 24 h. Averaged percentages were calculated and are shown within the dot plots followed by the ±SEM (*n* = 3). Asterisks indicate significant differences between treatments and 1% DMSO for each representative quadrant (* *p* < 0.05; ** *p* < 0.01 and *** *p* < 0.001). Co-detection of Annexin V (Ptd-L-Ser) and 7-AAD indicate apoptosis or necrosis, respectively. The red dots indicate cell populations within a sample. Cells in the live quadrant (**bottom left**) are viable, non-apoptotic and negative for Annexin V and 7-AAD. Cells in the early apoptotic quadrant (**bottom right**) are positive for Annexin V only, while the late apoptotic/dead quadrant (**top right**) represents cells that are positive for Annexin V and 7-AAD. The dead quadrant (**top left**) represents mostly nuclear debris and are cells positive for 7-AAD only.

In this study, PS was observed in the HT-29 cells treated with complexes **1** and **2** (Figure 5). The Annexin assay revealed that the number of apoptotic cells increased in a dose-dependent manner as the concentration of complex **1** increased. At 10 µM, more than half of the cell population remained viable, 62.97% (complex **1**) and 69.64% (complex **2**). However, at the 20 µM treatment, complex **1** resulted in a significant increase (*p* < 0.001) of 75.73% for the population undergoing late apoptosis, while complex **2** resulted in a lower apoptotic population of 38.75% (*p* < 0.05). Cells treated with 10 µM of the silver complexes resulted in a more prevalent apoptotic population than cells treated with 200 µM of CDDP (13.68%), while the vehicle control cells remained viable (90.43%). When compared with the positive necrotic H_2_O_2_ control, 94.76% of the population were significantly late apoptotic (*p* < 0.001). Similar results were seen by Altay et al., (2019) when MCF-7 breast cancer cells were treated with increasing concentrations of Ag(I) complexes [Ag_2_(μmef)_2_(2-pymet)_2_] and [Ag_2_(μmef)_2_(2-pyet)_2_] [39]. An increase in late apoptotic populations of up to 63.90% was observed as the concentration of the silver complexes increased from 12.5 µM to 50 µM. Ferreira et al. (2015), treated MCF-7 cells with various silver(I) thiocyanate-phosphine complexes [15]. A decrease in cellular viability and an increase in apoptotic mechanisms were observed. Di- and polynuclear silver(I) sac complexes with four various tertiary phosphane ligands have been studied by Yilmaz et al. (2014) [41]. These silver complexes showed greater anti-cancer activity when adjuvant with phosphine and were toxic to breast (MCF-7) and lung (A549) cells. These complexes resulted in IC_50_ concentrations larger in value than that of CDDP yet exhibited a strong inhibitory effect and selectivity towards MCF-7 cells. Dissimilar IC_50_ findings were seen with regards to complex **1** (Table 1), but a similar sustainable inhibitory effect towards HT-29 cells is evident.

**Figure 6:**
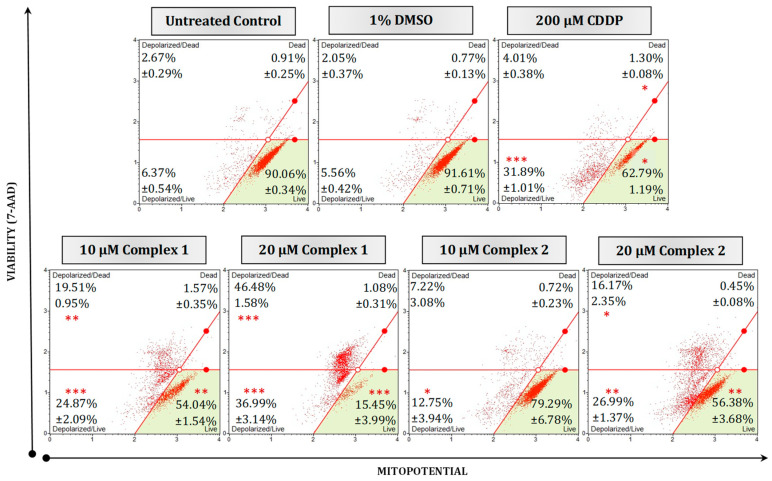
Mitochondrial potential and cellular plasma permeabilization were quantified in malignant HT-29 cells using a Muse^®^ MitoPotential kit. Cells were either left untreated or treated with 1% DMSO, 200 µM CDDP or 10 µM and 20 µM of selected complexes **1** and **2** for 24 h. Averaged percentages were calculated and are shown within the dot plots followed by the ±SEM (*n* = 3). Asterisks indicate significant differences between treatments and 1% DMSO for each representative quadrant (* *p* < 0.05; ** *p* < 0.01 and *** *p* < 0.001). Co-detection of MitoPotential dye and 7-AAD indicate intact mitochondria and cell death, respectively. The red dots indicate cell populations within a sample. Cells in the live quadrant (**bottom left**) are viable and have an intact mitochondrial membrane. These cells are negative for MitoPotential dye and 7-AAD. Cells in the depolarized/live quadrant (**bottom left**) are positive for MitoPotential dye only, indicating a depolarized mitochondrial membrane. The depolarized/dead quadrant (**top left**) represents cells that are positive for MitoPotential dye and 7-AAD, the structural integrity of these cells is damaged with a depolarized mitochondrial membrane. The dead quadrant (**top right**) represents dead cells with an intact mitochondrial membrane and are cells positive for 7-AAD only.

To further determine if the complexes’ apoptotic-inducing abilities were due to mitochondrial targeting, ΔΨm membrane potential and ROS production were studied. An increase in the production of ROS and depolarization of the ΔΨm have previously been linked to the signaling pathways that lead to apoptosis [42,43,44]. Any damage to the mitochondria or membrane integrity results in the release of apoptotic effectors which ultimately initiate the intrinsic or mitochondrial-mediated cell death cascade [40]. HT-29 cells were differentially treated for 24 h and subsequently subjected to a MitoPotential fluorescent dye and 7-AAD (a cell death marker). Figure 6 illustrates the impact on membrane integrity of different treatments. Untreated and vehicle control cells exhibited intact membranes, with live populations of 90.06% and 91.61%, respectively. In contrast, treatment with 20 µM of complex **1** caused a significant (*p* < 0.001) increase in membrane depolarization for live cells (36.99%) and dead cells (46.48%). Similarly, complex **2** displayed a notable (*p* < 0.01) increase in membrane depolarization of 26.99% at 20 µM, which was comparable to the effect observed at 10 µM but not as effective as that of complex **1**. Mitochondrial depolarization in malignant cell lines by silver complexes has been reported before [20,45]. Li et al., (2014) found that, when treating A549 lung cancer cells with the silver complex, Ag–SP–DNC, a concentration of 10 µM disrupted the ΔΨm following induced ROS generation [46].

**Figure 7:**
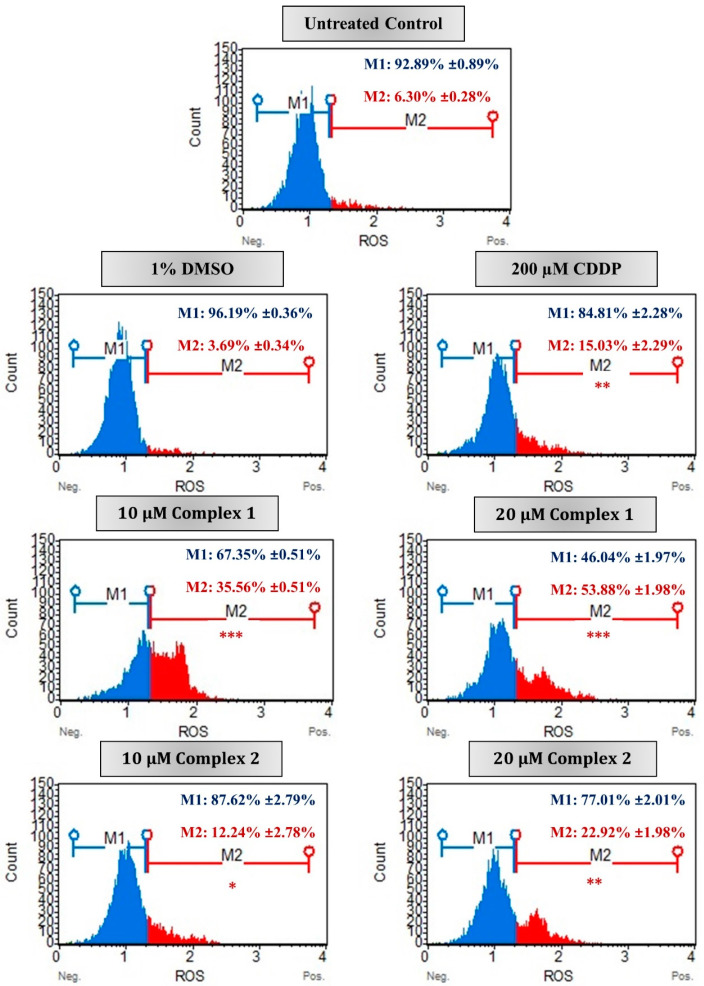
The ROS production in malignant HT-29 cells measured using the Muse^®^ Oxidative stress kit. Representative flow cytometric histograms are presented as untreated (control), treated with 1% DMSO (vehicle control), 200 µM CDDP (positive apoptotic control) or 10 µM and 20 µM of complexes **1** and **2** for 24 h. The percentages indicate average percentage of cellular populations that are either ROS negative (M1, blue) or ROS positive (M2, red) followed by the ±SEM (*n* = 3). Asterisks indicate significant differences between treatments and 1% DMSO for ROS-positive activity (* *p* < 0.05; ** *p* < 0.01 and *** *p* < 0.001).

In malignant cells, the excessive production of ROS results in the dysfunction of the mitochondria and numerous cellular components which in turn ultimately results in cell death [3,47]. In this study, the intracellular ROS production was monitored in the HT-29 cells after treatment with complex **1** and **2**. Flow cytometry data reveal that both complexes stimulated intracellular ROS in HT-29 cells in a dose-dependent manner after 24 h (Figure 7). The percentage of ROS+ (M2) cells increased from 35.56% to more than half of the population, 53.88%, after treatment with 10 µM and 20 µM of complex **1**. While for complex **2**, only 22.92% of the population was ROS+ after treatment with 20 µM. Differences remained significant when compared with the vehicle control cells, with 96.19% of the population ROS− (M1). Studies conducted by Al-kawmani et al. (2020), revealed a similar increase in intracellular ROS+ population in MCF-7 cells when treated with increasing concentrations of AgNPs after 24 h [48]. It was found that a higher concentration of AgNPs allowed for the elimination of antioxidants and the production of ROS, and ultimately DNA damage. Further confirmation was observed in Engelbrecht et al. (2018a), whose work has shown esophageal squamous carcinoma cells treated with 10 µM of the silver(I) thiocyanate complex [20]. This complex falls into the same phosphine silver monodentate family as complexes **1** and **2** used within this study [29]. In most of the cell population, 42.42% was ROS+ when compared with the vehicle control cells. This is comparable to the ROS+ population induced by complex **1** at 10 µM treatment. The above data are suggestive that treatment with complex **1** eventually leads to apoptosis in HT-29 cells via the production of ROS, decreased ΔΨm membrane potential and PS externalization.

## 4. Conclusions

Colorectal adenocarcinoma HT-29 cells were treated with two novel complexes: silver(I) diphenyl-2-pyridylphosphine]Br (**1**) and [silver(I) 4-(dimethylamino)phenyldiphenylphosphine]Br (**2**). Complex **1** resulted in a greater inhibitory effect, significantly targeting the mitochondria, and showed selectivity towards HT-29 cells. Complex **2** was less toxic in comparison with complex **1**, while both complexes resulted in minimal toxicity to non-malignant HEK-293 and MRHF cells. Complex **1** resulted in IC_50_ concentrations that were significantly less than CDDP after 24 h and 48 h, as well as morphological changes, PS externalization, increased levels of ROS and increased depolarization of the mitochondrial membrane. The observed changes collectively exhibit characteristics consistent with apoptosis. We hypothesize that complex **1** has the potential to be considered a metal-based candidate drug that could effectively target colorectal cancer.

## Figures and Tables

**Table 1:** The calculated IC_50_ concentrations for complex **1**, **2** and CDDP in malignant HT-29 cells, after 24 h and 48 h of treatment. The alamarBlue^®^ proliferation assay was used to determine cytotoxicity. The standard error of the mean (±SEM) is representative of at least nine independent experiments.

Treatment	**Malignant HT-29 Cells**	
	**IC_50_ Concentrations (±SEM)**	
	**24 h**	**48 h**
CDDP	200.96 µM (±10.35 µM)	55.16 µM (±3.46 µM)
1	7.49 µM (±0.20 µM)	5.92 µM (±0.29 µM)
2	21.75 µM (±1.97 µM)	185.28 µM (±5.19 µM)

## Data Availability

The data presented in this study are available on request from the corresponding author. The data are not publicly available due to limited access to secured and centralized ITS infrastructure.

## Supplementary Materials

The following supporting information can be downloaded at: https://www.mdpi.com/article/10.3390/biomedicines11102794/s1, Figure S1: Supplementary Information File_Complex **1**; Figure S2: Supplementary Information File_Complex **2**.

## Abbreviations

ΔΨm	Mitochondrial membrane potential
^13^C	Carbon 13 NMR
CDCl_3_	Deuterated chloroform
CDDP	Cis-diamine-dichloro platinum (cisplatin)
CHNS	Carbon, hydrogen, nitrogen, sulfur elemental analysis
DMEM	Dulbecco’s modified Eagles medium
DMSO	Dimethyl sulfoxide
FCS	Fetal calf serum
^1^H	Proton NMR
HBSS	Hanks’ balanced salt solution
IC_50_	Half maximal inhibitory concentration
IR	Infra-red spectrometry
NMR	Nuclear magnetic resonance spectrometry
^31^P	Phosphorous 31 NMR
PARP	Poly (ADP-ribose) polymerase
PBS	Phosphate buffered saline
PS	Phosphatidylserine
ROS	Reactive oxygen species
SEM	Standard error of the mean
