# Supplementary material for: Silver(I) Bromide Phosphines Induce Mitochondrial-Mediated Apoptosis in Malignant Human Colorectal Cells
Source: Biomedicines. 2023 Oct 14;11(10):2794. doi: 10.3390/biomedicines11102794 (PMC10604669; doi:10.3390/biomedicines11102794)
Supplement: Supplementary file 1 [file biomedicines-11-02794-s001.zip › Supplementary Information File_Complex 1.pdf]

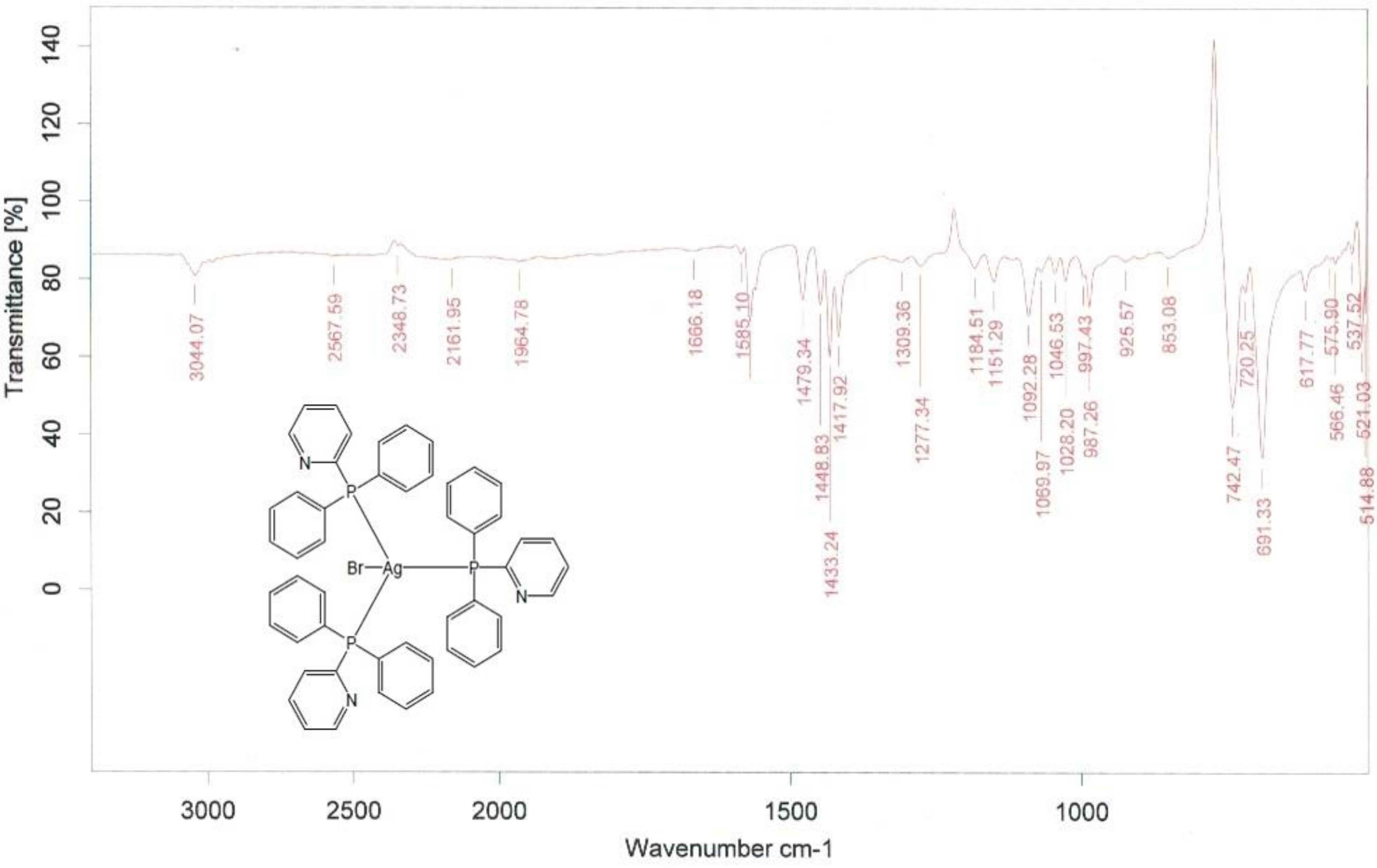

Transmittance [%]
140
120
100
80
60
40
20
0
3000
2500
2000
1500
1000
Wavenumber cm-1
3044.07
2567.59
2348.73
2161.95
1964.78
1666.18
1585.10
1479.34
1448.83
1433.24
1417.92
1309.36
1277.34
1184.51
1151.29
1092.28
1069.97
1046.53
1028.20
997.43
987.26
925.57
853.08
742.47
720.25
691.33
617.77
575.90
566.46
537.52
521.03
514.88
Br
Ag
P
N

102

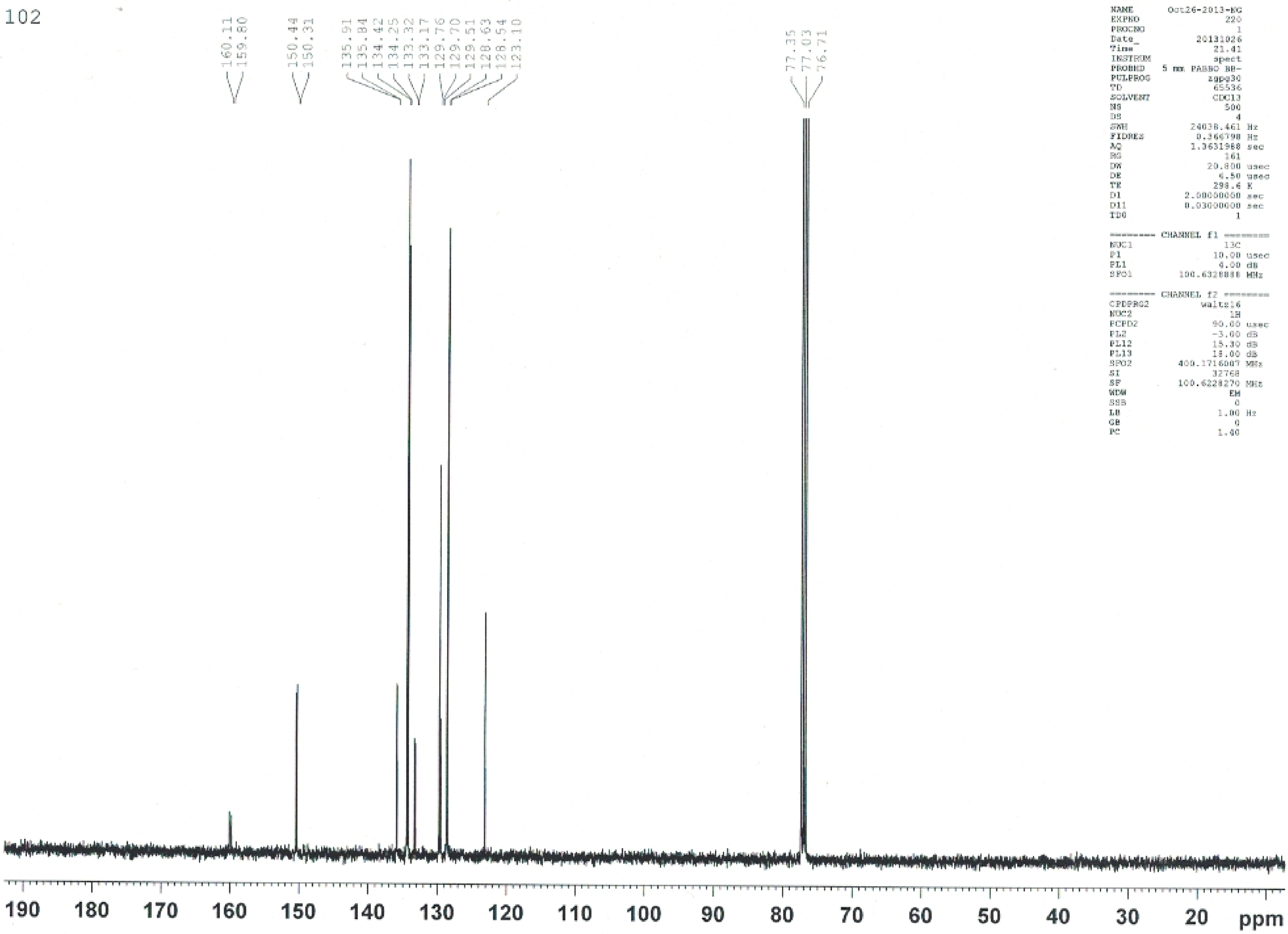

160.11
159.80
150.44
150.31
135.91
135.84
134.42
134.25
133.32
133.17
129.76
129.70
129.51
128.63
128.54
123.10
77.35
77.03
76.71
NAME Oct26-2013-BG
EXPNO 220
PROCNO 1
Date_ 20131026
Time 21.41
INSTRUM spect
PROBHD 5 mm PABBO BB-
PULPROG zgpg30
TD 65536
SOLVENT CDCl3
NS 500
DS 4
SWH 24038.461 Hz
FIDRES 0.366798 Hz
AQ 1.3631988 sec
RG 161
DW 20.800 usec
DE 6.50 usec
TE 298.6 K
D1 2.00000000 sec
D11 0.03000000 sec
TD0 1
======== CHANNEL f1 ========
NUC1 13C
P1 10.00 usec
PL1 4.00 dB
SFO1 100.6328088 MHz
======== CHANNEL f2 ========
CPDPRG2 waltz16
NUC2 1H
PCPD2 90.00 usec
PL2 -3.00 dB
PL12 15.30 dB
PL13 18.00 dB
SFO2 400.1716007 MHz
SI 32768
SF 100.6228270 MHz
WDW EM
SSB 0
LB 1.00 Hz
GB 0
PC 1.40
190 180 170 160 150 140 130 120 110 100 90 80 70 60 50 40 30 20 ppm

102

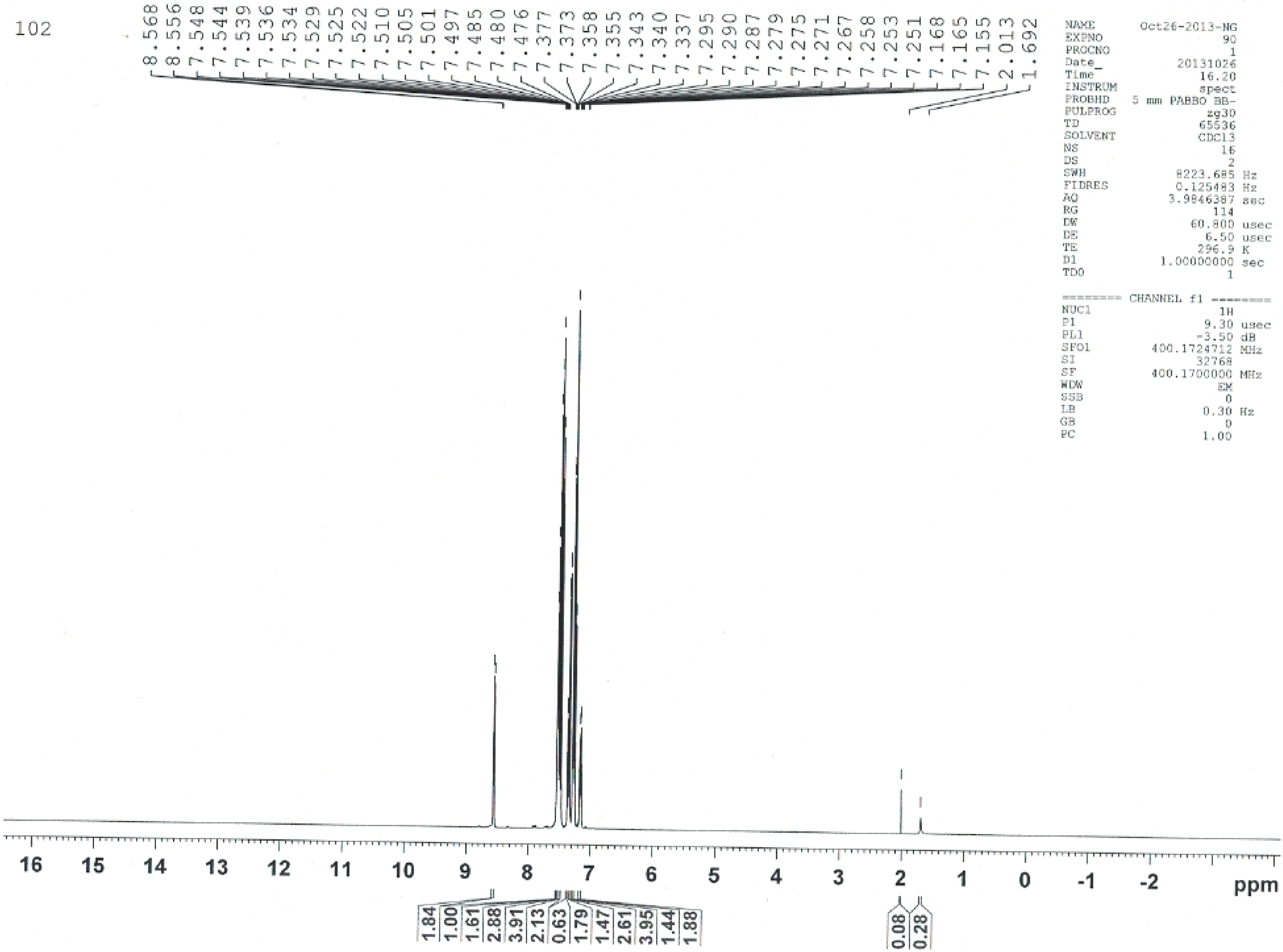

NAME	Oct26-2013-NG
EXPNO	90
PROCNO	1
Date_	20131026
Time_	16.20
INSTRUM	spect
PROBHD	5 mm PABBO BB-
PULPROG	zg30
TD	65536
SOLVENT	CDCl3
NS	16
DS	2
SWH	8223.685 Hz
FIDRES	0.125483 Hz
AQ	3.9846387 sec
RG	114
DW	60.800 usec
DE	6.50 usec
TE	296.9 K
D1	1.00000000 sec
TD0	1

======== CHANNEL f1 ========

NUC1	1H
P1	9.30 usec
PL1	-3.50 dB
SFO1	400.1724712 MHz
SI	32768
SF	400.1700000 MHz
WDW	EM
SSB	0
LB	0.30 Hz
GB	0
PC	1.00

102

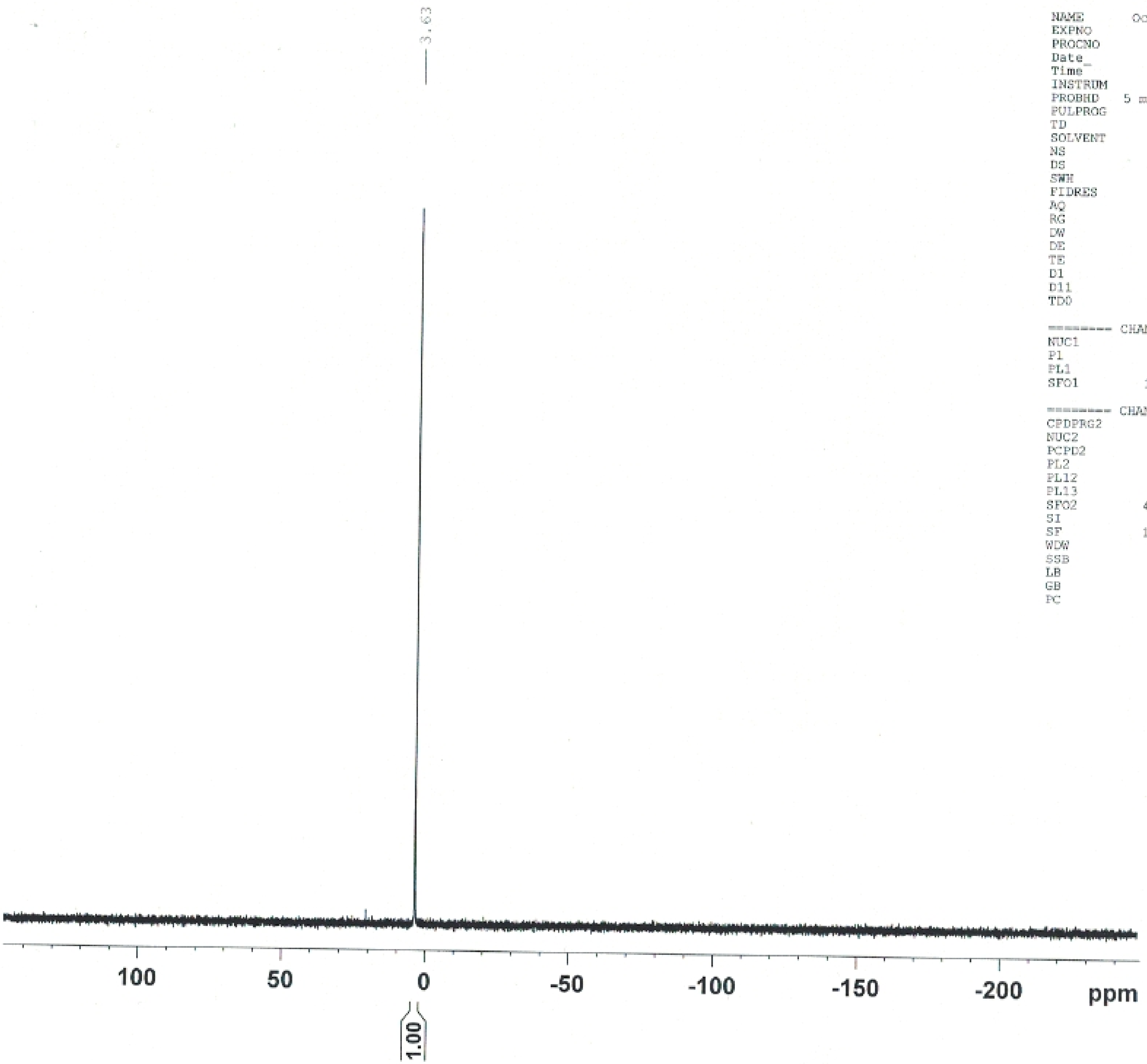

NAME	Oct26-2013-NG	
EXPNO	91	
PROCNO	1	
Date_	20131026	
Time	16.22	
INSTRUM	spect	
PROBHD	5 mm PABBO BB-	
PULPROG	zgpg30	
TD	65536	
SOLVENT	CDCl3	
NS	16	
DS	4	
SWH	64102.563	Hz
FIDRES	0.978127	Hz
AQ	0.5112308	sec
RG	2050	
DW	7.800	usec
DE	6.50	usec
TE	297.6	K
D1	2.00000000	sec
D11	0.03000000	sec
TD0	1	
======== CHANNEL f1 ========		
NUC1	31P	
P1	9.20	usec
PL1	0.00	dB
SFO1	161.9836857	MHz
======== CHANNEL f2 ========		
CPDPRG2	waltz16	
NUC2	1H	
PCPD2	90.00	usec
PL2	-3.00	dB
PL12	15.30	dB
PL13	18.00	dB
SFO2	400.1716007	MHz
SI	32768	
SF	161.9917850	MHz
WDW	EM	
SSB	0	
LB	1.00	Hz
GB	0	
PC	1.40	
