# Supplementary material for: Silver(I) Bromide Phosphines Induce Mitochondrial-Mediated Apoptosis in Malignant Human Colorectal Cells
Source: Biomedicines. 2023 Oct 14;11(10):2794. doi: 10.3390/biomedicines11102794 (PMC10604669; doi:10.3390/biomedicines11102794)
Supplement: Supplementary file 1 [file biomedicines-11-02794-s001.zip › Supplementary Information File_Complex 2.pdf]

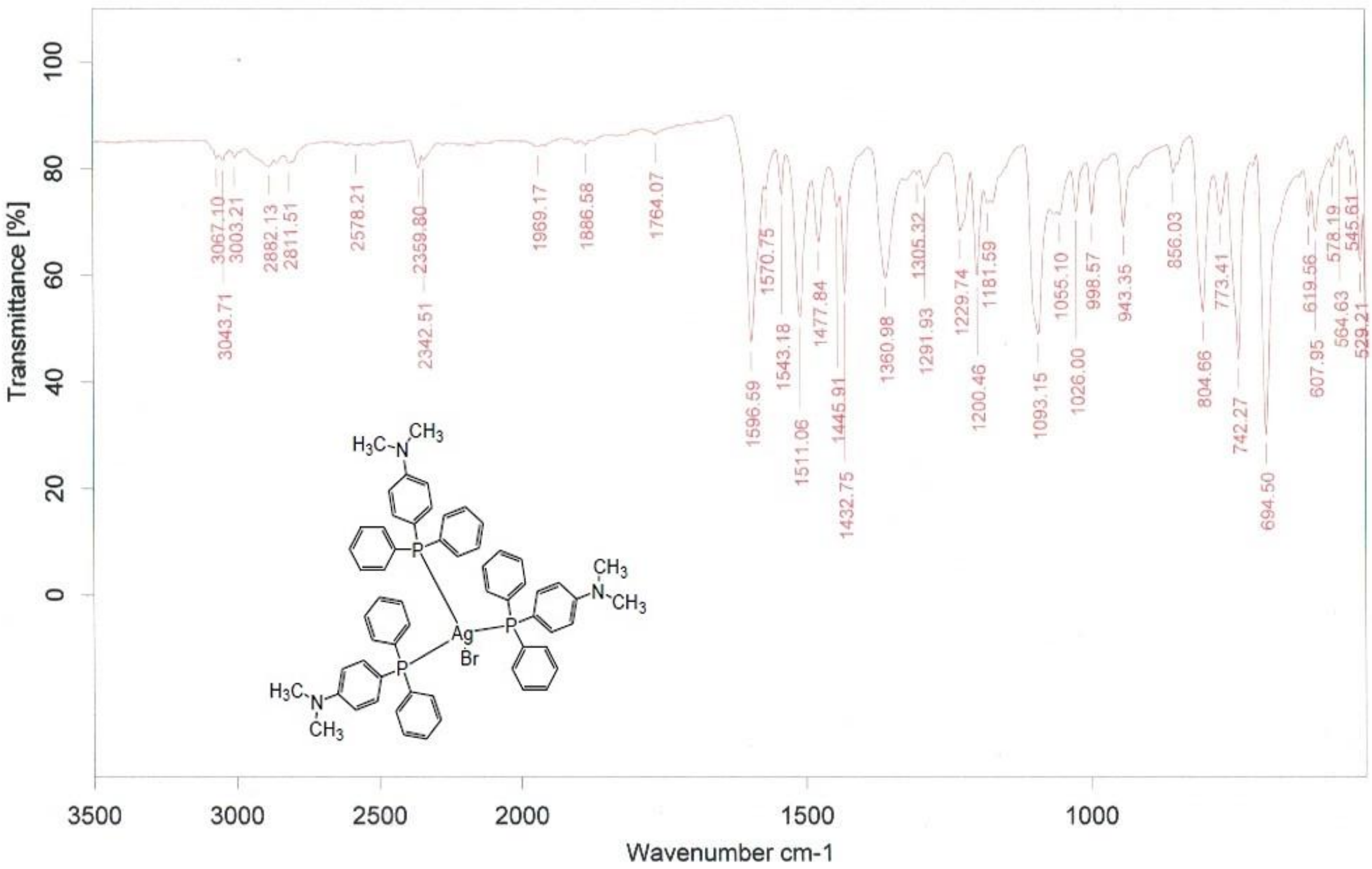
Transmittance [%]
Wavenumber cm-1
3067.10
3043.71
3003.21
2882.13
2811.51
2578.21
2359.80
2342.51
1969.17
1886.58
1764.07
1596.59
1570.75
1543.18
1511.06
1477.84
1445.91
1432.75
1360.98
1305.32
1291.93
1229.74
1200.46
1181.59
1093.15
1055.10
1026.00
998.57
943.35
856.03
804.66
773.41
742.27
694.50
619.56
607.95
578.19
564.63
545.61
529.21

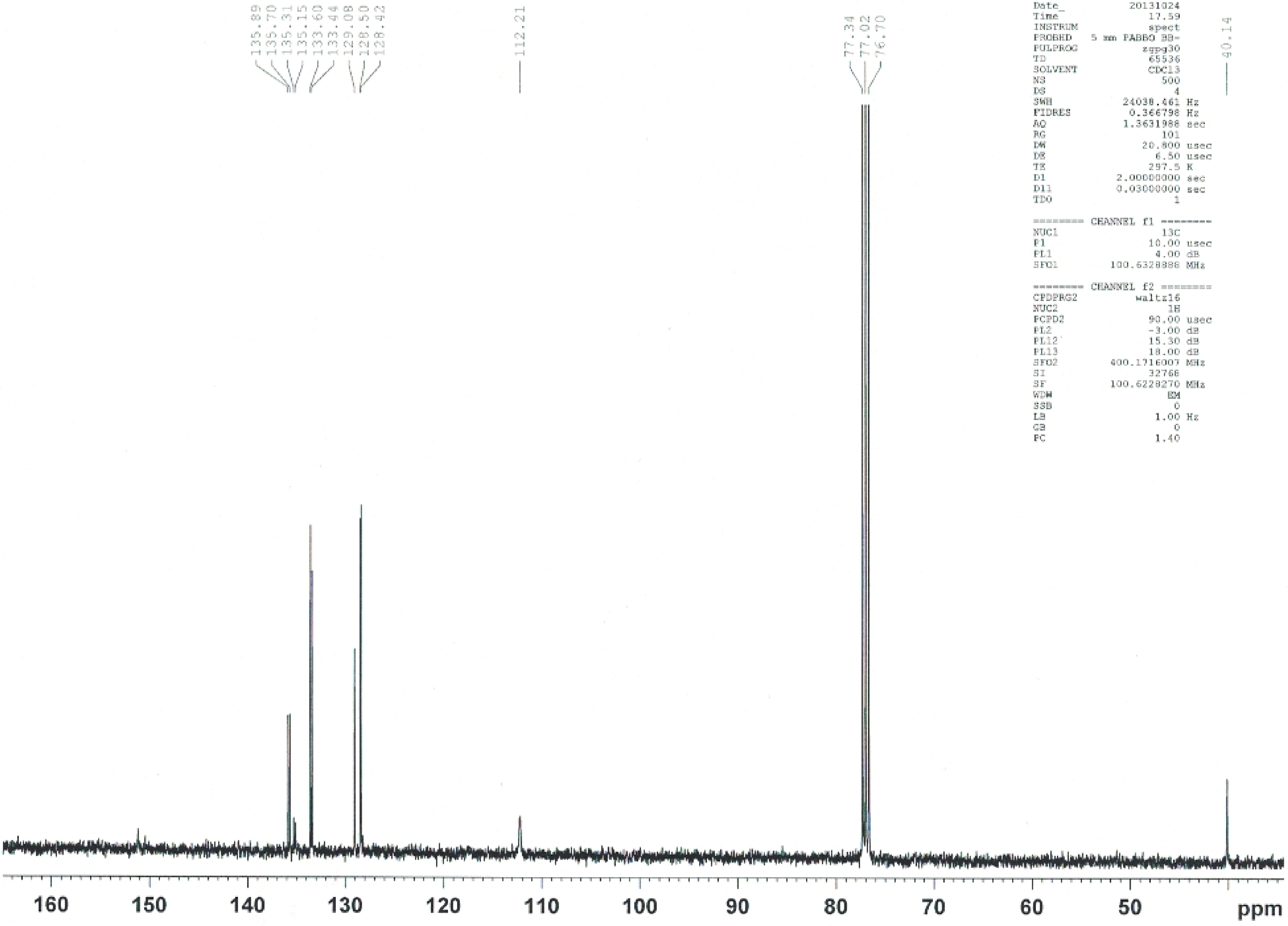


```
NAME         Oct24-2013-NG
EXPNO                   32
PROCNO                   1
Date_             20131024
Time                 17.59
INSTRUM              spect
PROBHD   5 mm PABBO BB-
PULPROG            zgpg30
TD                   65536
SOLVENT              CDCl3
NS                     500
DS                       4
SWH              24038.461 Hz
FIDRES            0.366798 Hz
AQ               1.3631988 sec
RG                     101
DW                  20.800 usec
DE                    6.50 usec
TE                   297.5 K
D1              2.00000000 sec
D11             0.03000000 sec
TD0                      1

======== CHANNEL f1 ========
NUC1                   13C
P1                   10.00 usec
PL1                   4.00 dB
SFO1           100.6328886 MHz

======== CHANNEL f2 ========
CPDPRG2            waltz16
NUC2                    1H
PCPD2                90.00 usec
PL2                  -3.00 dB
PL12                 15.30 dB
PL13                 18.00 dB
SFO2           400.1716007 MHz
SI                   32768
SF             100.6228270 MHz
WDW                     EM
SSB                      0
LB                    1.00 Hz
GB                       0
PC                    1.40
```

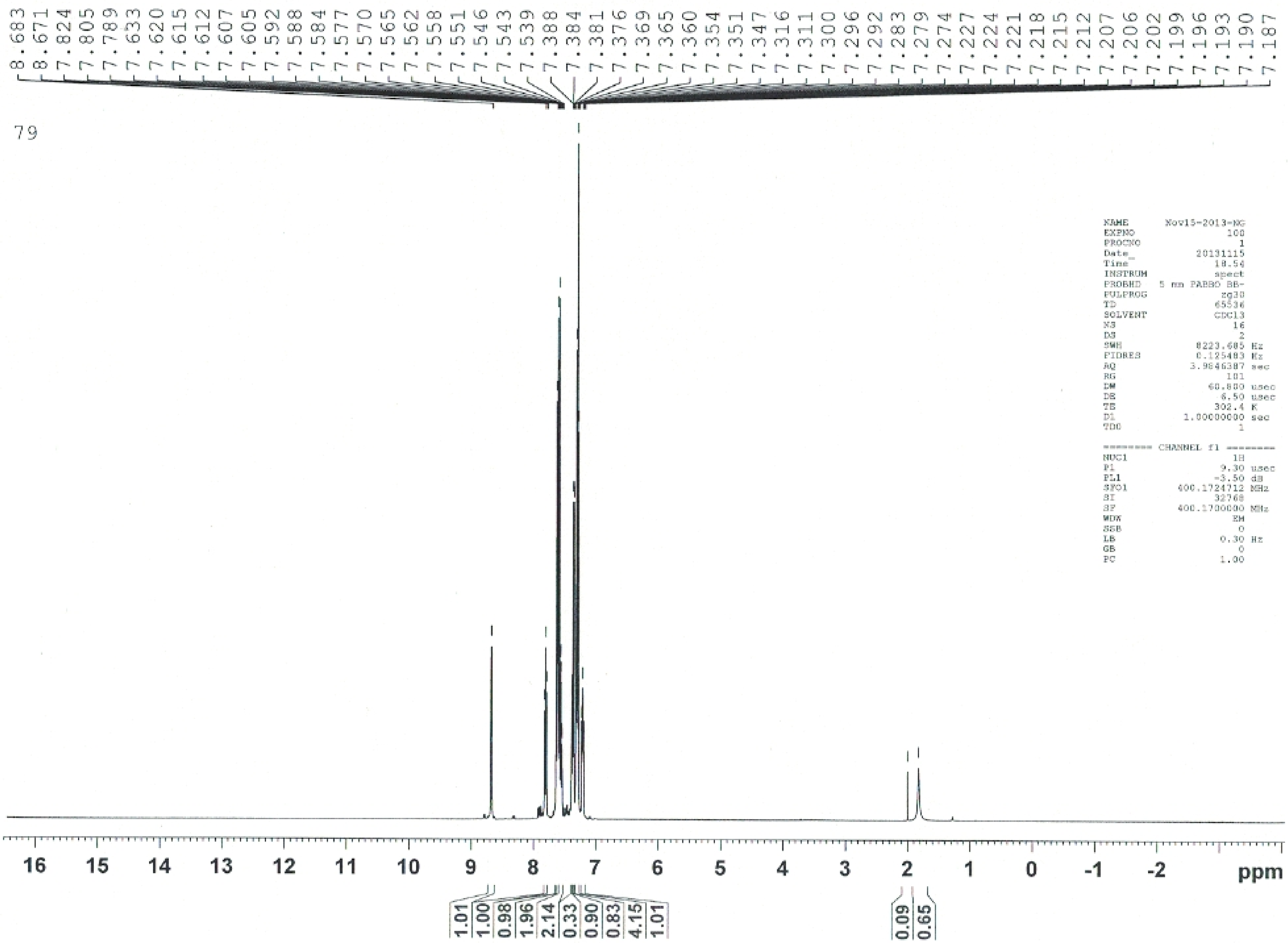
8.683
8.671
7.824
7.805
7.789
7.633
7.620
7.615
7.612
7.607
7.605
7.592
7.588
7.584
7.577
7.570
7.565
7.562
7.558
7.551
7.546
7.543
7.539
7.388
7.384
7.381
7.376
7.369
7.365
7.360
7.354
7.351
7.347
7.316
7.311
7.300
7.296
7.292
7.283
7.279
7.274
7.227
7.224
7.221
7.218
7.215
7.212
7.207
7.206
7.202
7.199
7.196
7.193
7.190
7.187
79
NAME Nov15-2013-NG
EXPNO 100
PROCNO 1
Date_ 20131115
Time 18.54
INSTRUM spect
PROBHD 5 mm PABBO BB-
PULPROG zg30
TD 65536
SOLVENT CDCl3
NS 16
DS 2
SWH 8223.685 Hz
FIDRES 0.125483 Hz
AQ 3.9846387 sec
RG 101
DW 60.800 usec
DE 6.50 usec
TE 302.4 K
D1 1.00000000 sec
TD0 1
======== CHANNEL f1 ========
NUC1 1H
P1 9.30 usec
PL1 -3.50 dB
SFO1 400.1724712 MHz
SI 32768
SF 400.1700000 MHz
WDW EM
SSB 0
LB 0.30 Hz
GB 0
PC 1.00
16
15
14
13
12
11
10
9
8
7
6
5
4
3
2
1
0
-1
-2
ppm
1.01
1.00
0.98
1.96
2.14
0.33
0.90
0.83
4.15
1.01
0.09
0.65

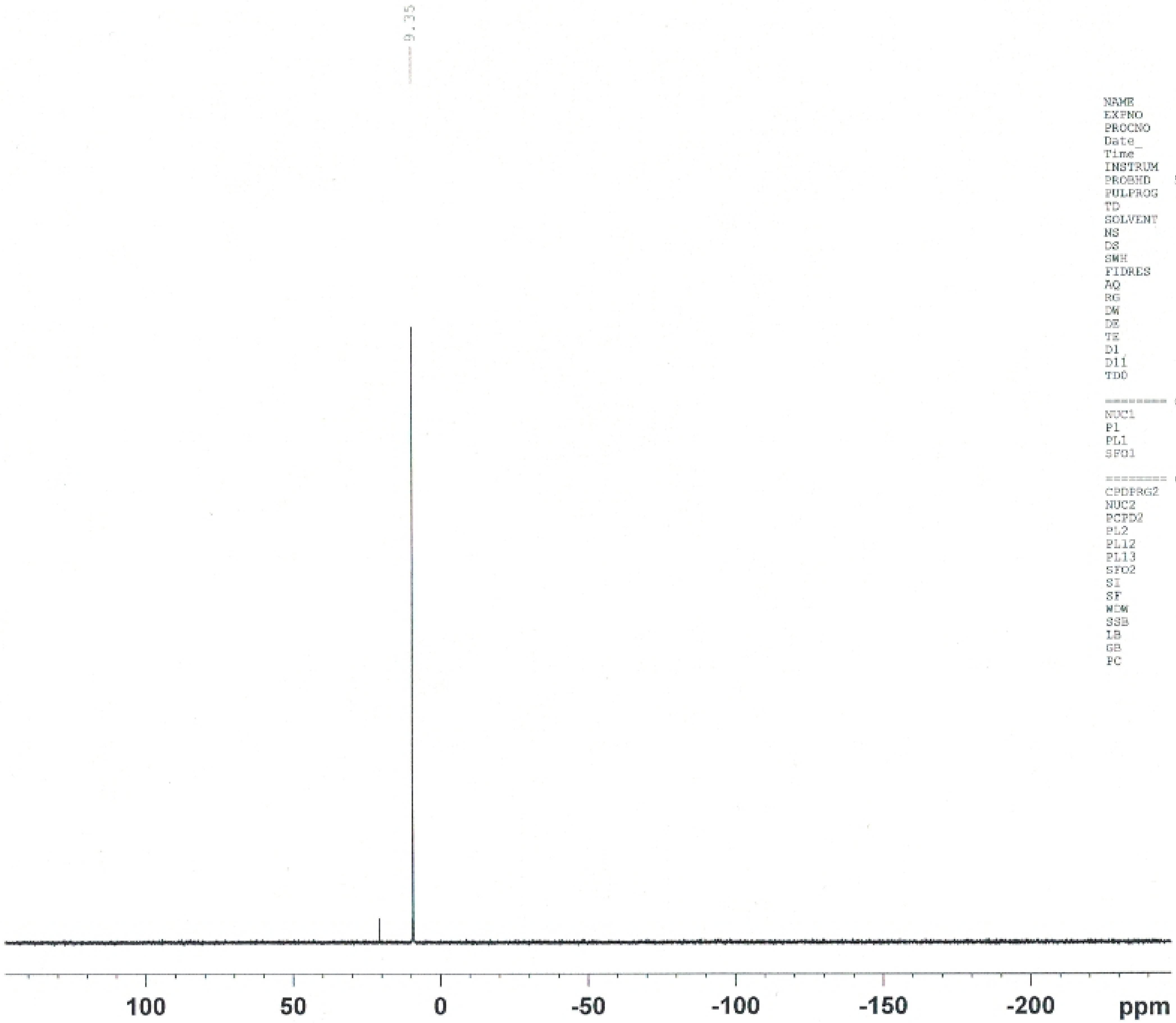


```
NAME       Nov15-2013-NG
EXPNO                101
PROCNO                 1
Date_           20131115
Time               18.57
INSTRUM            spect
PROBHD   5 mm PABBO BB-
PULPROG          zgpg30
TD                 65536
SOLVENT            CDCl3
NS                    16
DS                     4
SWH           64102.563 Hz
FIDRES         0.978127 Hz
AQ            0.5112308 sec
RG                  2050
DW                 7.800 usec
DE                  6.50 usec
TE                 303.2 K
D1           2.00000000 sec
D11          0.03000000 sec
TD0                    1

======== CHANNEL f1 ========
NUC1                 31P
P1                  9.20 usec
PL1                 0.00 dB
SFO1        161.9836857 MHz

======== CHANNEL f2 ========
CPDPRG2          waltz16
NUC2                  1H
PCPD2              90.00 usec
PL2                -3.00 dB
PL12               15.30 dB
PL13               18.00 dB
SFO2         400.1716007 MHz
SI                 32768
SF           161.9917850 MHz
WDW                   EM
SSB                    0
LB                  1.00 Hz
GB                     0
PC                  1.40
```
